# Forest Fine Root Litter Mitigates the NH_3_ Volatilization and N_2_O Emission from N-Applied Agriculture Soil

**DOI:** 10.3390/plants15010057

**Published:** 2025-12-24

**Authors:** Si Wu, Lei Chu, Guanglong Zhu, Lihua Ning

**Affiliations:** 1 Co-Innovation Center for Sustainable Forestry in Southern China, College of Forestry and Grassland, Nanjing Forestry University, Nanjing 210037, China; nlwus@njfu.edu.cn; 2 Maize Research Centre, Jiangsu Academy of Agricultural Sciences, Nanjing 210014, China; 3 Joint International Research Laboratory of Agriculture and Agri-Product Safety, The Ministry of Education of China, Yangzhou University, Yangzhou 225009, China; g.zhu@yzu.edu.cn

**Keywords:** crop yield, forest fine root, NH_3_ loss, N_2_O emission, soil properties

## Abstract

Forest fine root litter enters agricultural soils in some cases and its decomposition would change the soil’s properties. However, how this process further influences the ammonia (NH_3_) volatilization and nitrous oxide (N_2_O) emission from agricultural soil receiving fertilizer nitrogen (N) is unknown. Here, we conducted a soil pot experiment to investigate the responses of the aforementioned gaseous N losses during wheat season to fine root litters derived from *Populus deltoides* (RP) and *Metasequoia glyptostroboides* (RM) incorporations. The results showed that two forest fine root litters reduced total NH_3_ losses by 30.6−31.9% from 180 kg N ha^−1^ applied to farmland soil, and this effect was attributed to decreased soil urease activity and ammonium-N during the basal N fertilization period. Whether receiving fertilizer N or not, N_2_O emissions from farmland soil were significantly (*p* < 0.05) mitigated by 62.8–68.2% and 43.0−50.0% following the RP and RM incorporation, respectively. Lower N_2_O emission was ascribed to increased soil pH but decreased soil nitrate-N and bulk density. In addition, less AOA and AOB *amoA* but more *nosZ* gene abundances explained the fine root litter-induced N_2_O mitigation effect. Neither forest fine root litter exerted a negative effect on wheat grain yield and crop N use efficiency in N-added agriculture soil. In conclusion, forest fine root litter incorporation could help to mitigate gaseous N losses via NH_3_ volatilization and N_2_O emission from fertilizer-N-applied agricultural soils, and without crop production loss.

## 1. Introduction

Inorganic nitrogen (N) fertilizer application is an important basis management for stable crop production [1]. However, excessive N fertilizer input not only decreases N use efficiency (NUE) but also threatens environmental sustainability, especially increased reactive N losses via ammonia (NH_3_) volatilization and nitrous oxide (N_2_O) emission [2,3]. The aforementioned gaseous N losses play a dominant role in atmospheric particulate matter (PM_2.5_) pollution and global warming potential (GWP), respectively [4]. Therefore, to reduce NH_3_ and N_2_O losses is of great significance in agricultural N management, in particular, clarifying the factors that decide the NH_3_ and N_2_O emission loads from varied ecosystems.

Agroforestry intercropping is considered as an effective approach for sustainable land use and has been widely spread to reduce water, soil, and nutrient losses from agricultural soil, especially in temperate and tropical areas [5,6,7]. Globally, there are approximately 1.6 billion hectares of agroforestry systems globally [8,9], which will be further expanded in the future to resolve the land conflict between timber production and agricultural food security [10]. In China, agroforestry intercropping practices are conducted with approximately 2% of the total arable land [11], with more evident ecological benefits including enhancing the resistance of crops to severe weather, increasing crop productivity, modulating the regional micro-climate, reducing soil erosion, mitigating nutrient losses, such as N loss, and improving NUE [5,12]. Furthermore, Singh et al. (2024) found that the choice of tree species also had various impacts on crop production in agroforestry intercropping ecosystems (the wheat yield of the poplar system was 44.1% higher than that of the eucalyptus system) [12]. Therefore, considering the tree species of agroforestry intercropping is also important to crop productivity.

There were only a few studies focused on the NH_3_ and N_2_O emissions in the agroforestry intercropping systems. For example, Cheng et al. (2013) demonstrated that cumulative NH_3_ volatilization of poplar–wheat intercropping was 10.06–16.83 kg ha^−1^ [13]. Ansari et al. (2023) found that the agroforestry system exerted significantly lower average N_2_O flux (0.70 kg ha^−1^ yr^−1^) than the corn–soybean intercropping system (2.16 kg ha^−1^ yr^−1^) [14]. Therefore, data on gaseous N losses via NH_3_ volatilization and N_2_O emission from agroforestry intercropping ecosystems need to be enriched. Particularly, it remains to be explored how the fine root litters derived from forest plants affect soil N emissions during agricultural crop production.

Forest fine roots, defined as those with a diameter of no more than 2 mm, are vital organs for resource acquisition and nutrient input [15]. The competition for resources between crops and tree fine roots leads to lower crop yield in near-forest zones, which limits the productivity of agroforestry intercropping ecosystems [16]. This limitation refers to the reduction effect of living forest fine roots on the crop grains. Nevertheless, there is still abundant forest fine root litter delivered to the tree–crop interface soil of agroforestry intercropping ecosystems during the management of farmland like ploughing and fine roots turnover [17,18]. These forest fine root litters usually suffer from decomposition and decay, accompanied by nutrient return, the material cycle, and the alteration of soil properties [19], which may exert a positive effect on crop production and soil N emissions.

Earlier studies found that fine root litter decomposition was related to soil urease, pH, moisture, temperature, or cation exchange capacity probably by mediating the NH_3_ volatilization process [20,21]. In addition, the nutrient input from forest litter stimulates soil microbial biomass and activity, encouraging changes in the abundance of representative functional genes, and then regulates soil N_2_O emissions by altering N cycle functional genes (i.e., AOA and AOB *amoA*, *nirK*, *nirZ*, and *nosZ*) [22,23,24]. Therefore, the influence of fine root litter on soil N emissions might be affected indirectly by changes in these soil properties in agroforestry regions [25]. Furthermore, fine root litter decomposition was closely related to exogenous N addition and various tree species [26]. A bag-burying experiment based on a single exponential model suggested that poplar and metasequoia exert remarkably different decomposition coefficients of 0.22–0.25 and 0.54–0.70, respectively, under stimulated N deposition with 5–30 g m^−2^ yr^−1^ [27]. Mixed N fertilization promotes fine root litter decomposition rates and N input accelerates the fine root turnover rate by 27.8% in the surface soil [28,29]. Hence, it is of great importance to consider the fine root litter types/species and N addition when exploring how forest fine root litters impact NH_3_ volatilization and N_2_O emissions.

During ploughing and natural decomposition, fine root litters from the forest underground enter the farmland to create a relatively new soil environment in the agroforestry ecosystem [18,30]. This experiment simulated this situation by exogenously adding dead forest fine root litter into farmland soil. Here, we hypothesized that forest fine root litters change the N losses via NH_3_ volatilization and N_2_O emission, and this effect is dependent on root type. Therefore, we conducted a soil pot experiment with two fine root litters (i.e., *Populus deltoides* and *Metasequoia glyptostroboides*) under non-fertilized and fertilized N treatments, in order to investigate the responses of NH_3_ volatilization, N_2_O emissions, and wheat yield to forest fine root litter from the tree–crop interface soil of an agroforestry system, and preliminarily clarify the underlying mechanisms. The findings of our study could provide essential theoretical and technical supports for sustainably managing the agroforestry system and mitigating N losses for cleaner agroforestry production.

## 2. Results

### 2.1. NH_3_ Volatilization

**Figure 1:**
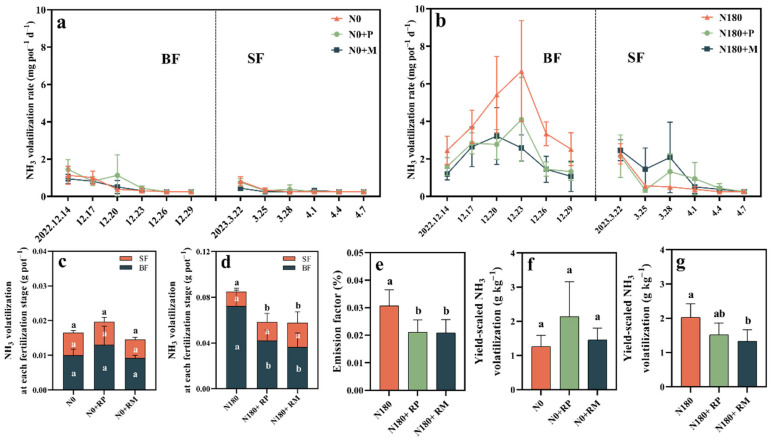
Effects of forest fine root litters of *Populus deltoides* (RP) and *Metasequoia glyptostroboides* (RM) on ammonia (NH_3_) volatilization farmland soils with 0 (N0) and 180 kg N ha^−1^ (N180) application. Dynamics of NH_3_ volatilization rate within 18 days after N fertilization (**a**,**b**). Cumulative NH_3_ volatilization at the basal (BF) and supplementary (SF) N fertilization stage (**c**,**d**). NH_3_ volatilization emission factor (**e**) and the yield-scaled NH_3_ volatilization (**f**,**g**). Different lowercase letters indicate the statistically significant differences between the treatments under the same N application level by Duncan’s method at *p* < 0.05.

The daily NH_3_ volatilization rate recorded under the no-N treatments was very low with less than 2 mg pot^−1^ d^−1^ (Figure 1a). When the N-applied rate was 180 kg N ha^−1^, the NH_3_ volatilization rate first sharply increased and then decreased to a steady level following the basal fertilization (BF) (Figure 1b). The peak NH_3_ volatilization rates that were observed on the 9–12th day after BF reached up to 3.21–6.68 mg pot^−1^ d^−1^ under three N-amended treatments. The NH_3_ volatilization rates of the two treatments with fine root litter amendments were generally lower than those without fine root litters during the whole observation of BF. At the SF observation, the fluctuation ranges among N180, N180+RP, and N180+RM were small.

RP and RM had no significant (*p* < 0.05) influence on total and yield-scale NH_3_ volatilization from no-urea-N-applied soils (Figure 1c,f). Interestingly, the total NH_3_ volatilization and emission factor from N180+RP and N180+RM were, on average, 31.3% and 31.6% lower than those from the N180 treatment, respectively (Figure 1d,e). Of these, the 41.7−49.6% significant (*p* < 0.05) reduction efficiencies in NH_3_ volatilization resulting from the forest fine root litter were mainly recorded during BF observation (Figure 1d). In particular, N180+RM had a 34.5% significantly (*p* < 0.05) lower yield-scaled NH_3_ volatilization than N180 (Figure 1g).

### 2.2. Soil N_2_O Emission

**Figure 2:**
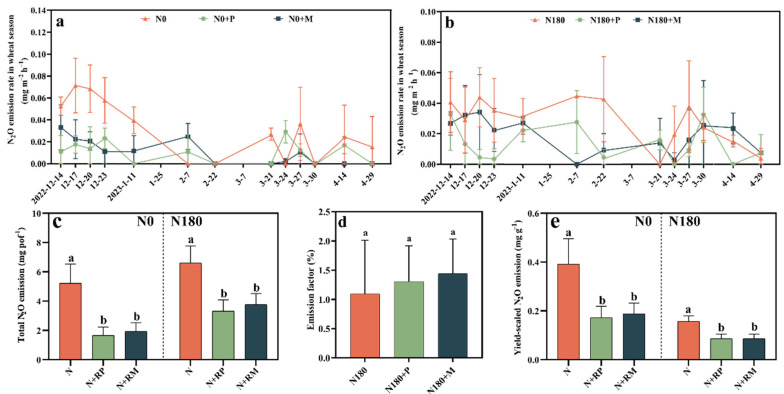
Effects of forest fine root litters of *Populus deltoides* (RP) and *Metasequoia glyptostroboides* (RM) on the nitrous oxide (N_2_O) emission from 0 (N0) and 180 kg N ha^−1^ (N180) applied farmland soils. Dynamics of N_2_O emission rate during the whole wheat season (**a**,**b**). Total N_2_O emission (**c**). N_2_O emission factor (**d**) and yield-scaled N_2_O emission (**e**). Different lowercase letters indicate that the differences between the treatments with the same N application rate were statistically significant by Duncan’s method at *p* < 0.05.

Without N fertilizer input, the N_2_O emission rates in N0+RP and N0+RM treatments were always less than that in the N0 treatment during the first five observations. Although the N_2_O emission rates demonstrated a fluctuating trend during other observations throughout the wheat growth cycle, the average N_2_O emission rates of fine root litter-added treatments were lower than those of no treatment. (Figure 2a). As for the 180 kg N ha^−1^ amendment, the N_2_O emission rates with fine root litter amendment were lower than those with no fine root litter added during most of the wheat growth observations (Figure 2b). Therefore, the average N_2_O emission rates in N180+RP and N180+RM (0.013 and 0.018 mg m^−2^ h^−1^) were both lower than that in N180 (0.028 mg m^−2^ h^−1^) during the whole wheat growth period.

Both forest fine root litters significantly (*p* < 0.05) mitigated total N_2_O emission and yield-scaled N_2_O emission regardless of N application, especially in RP treatment (Figure 2c,e). Of these, RP and RM reduced total N_2_O emissions by 62.8–68.2% and 43.0–50.0% under 0 and 180 kg ha^−1^ N inputs, respectively (Figure 2c). Correspondingly, the two-forest fine root litter significantly (*p* < 0.05) mitigated the yield-scaled N_2_O emission by 45.0–56.0% under two N inputs (Figure 2e). However, either RP or RM changed the N_2_O emission factor in farmland soil, with that of N180+RP and N180+RM being relatively higher than that in N180, but the difference was not statistically significant (Figure 2d).

### 2.3. Soil Properties and Functional Gene Copies Related to N Cycling

#### 2.3.1. Soil Properties

**Figure 3:**
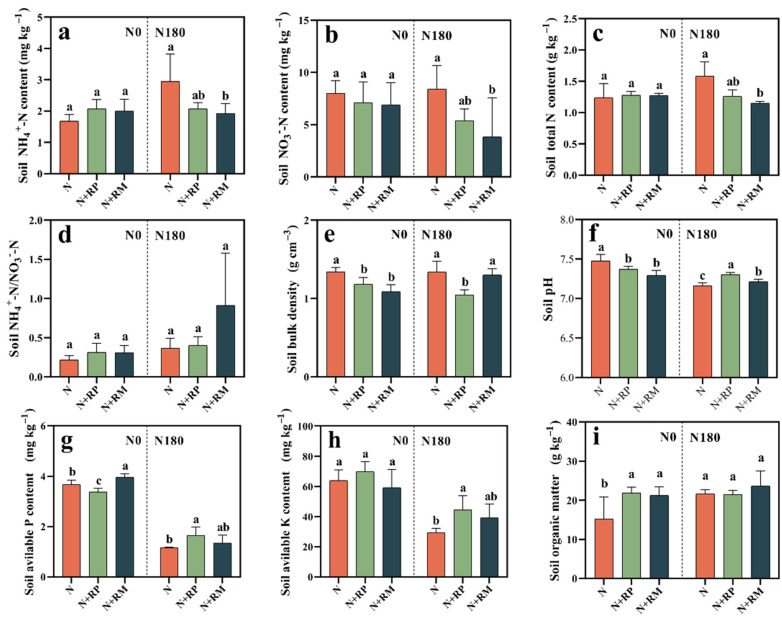
Effects of forest fine root litters of *Populus deltoides* (RP) and *Metasequoia glyptostroboides* (RM) on properties of 0−10 cm topsoils from farmland with 0 (N0) and 180 kg N ha^−1^ (N180) application. Soil NH_4_^+^-N (**a**), NO_3_^−^-N (**b**), total N (**c**), NH_4_^+^-N/NO_3_^−^-N (**d**), bulk density (**e**), pH (**f**), available P (**g**), available K (**h**), and soil organic matter (**i**). Different lowercase letters indicate the significant differences between the treatments under the same N application level by Duncan’s method at *p* < 0.05.

Under the no-fertilizer-N-added treatment, fine root litters did not change the soil NH_4_^+^-N, NO_3_^−^-N, and total N contents or the NH_4_^+^-N/NO_3_^−^-N (Figure 3a–d). Nevertheless, both RP and RM significantly reduced the aforementioned first three indices under 180 kg N ha^−1^, especially in N180+RM, and these were 34.6%, 54.5%, and 10.9% (*p* < 0.05) lower than in N180 alone.

Forest fine root litters reduced the bulk density of 0−10 cm topsoil after harvest at the same N application level (Figure 3e). The soil bulk density in N0+RP and N0+RM significantly (*p* < 0.05) decreased by 11.9 and 18.7%, compared to N0 alone, respectively. At the same time, the bulk density of N180+RP was 21.6% significantly (*p* < 0.05) lower than that of N180.

RP and RM significantly (*p* < 0.05) decreased the soil pH by 0.11−0.18 units without N input, while they significantly (*p* < 0.05) increased it by 0.15 and 0.06 units when 180 kg N ha^−1^ was applied (Figure 3f).

Compared with N0 alone, N0+RP decreased available P by 7.9%, while N0+RM increased it by 7.9% (Figure 3g). Both fine root litters exerted little influence on the available K with no N fertilizer (Figure 3h). In addition, both RP and RM increased the available P and available K when we applied fertilizer at a rate of 180 kg ha^−1^ into experimental soil. Specifically, N180+RP treatment significantly (*p* < 0.05) increased the soil available P and K contents by 41.0% and 51.6%, respectively. Furthermore, two fine root litters significantly (*p* < 0.05) increased soil organic matter (SOM) by 39.2−43.6% with no N fertilizer input, while they had no significant effect on SOM under 180 kg N ha^−1^ applied to the soil (Figure 3i).

#### 2.3.2. Functional Gene Copies Related to N Cycling

During the BF, fine root litter neither altered the gene copies of AOB *amoA* and *nirK*, nor did they impact the (*nirS*+*nirK*)/*nosZ* ratio when no fertilizer-N was applied (Table 1). The *nosZ* gene copies of N0+RP and N0+RM increased by 32.5–61.7%, compared to N0 alone. When 180 kg N ha^−1^ was added to the soil, RP significantly (*p* < 0.05) decreased the gene copies of AOA and AOB *amoA* by 26.0% and 23.4%, respectively. Up until the SF, fine root litter decreased the AOA *amoA* gene copies regardless of whether N fertilizer was applied (Table 1). The gene copies of AOB *amoA*, *nirK*, *nirS*, and *nosZ* as well as the (*nirS*+*nirK*)/*nosZ* ratio all exerted an increasing trend with fine root litter under the no-N-added soil. In particular, the *nosZ* gene copies of N0+RM and N0+RP were 9.5% and 46.6% higher than that of N0 alone. When the N application rate reached 180 kg N ha^−1^, the two fine root litters decreased AOB *amoA* gene copies by 12.3–34.2%, compared to N180 alone. Meanwhile, the *nosZ* gene copies with fine root litter were 33.9–67.8% higher than that without fine root litter.

### 2.4. Wheat Growth, Yield, and NUE

#### 2.4.1. Plant Height and Flag Leaf SPAD

With no urea N application, both forest fine root litters significantly (*p* < 0.05) lowered the wheat plant height at earing and maturation phases by 5.3–5.5% and 6.6–6.8%, respectively, and decreased the leaf SPAD at earing stage by 21.1–23.7%, compared to N0 alone. Nevertheless, fine root litter exerted no remarkable influence on the plant height and SPAD of wheat planted in soils with 180 kg N ha^−1^ at each growth stage (Figure S1).

#### 2.4.2. Wheat Yield, N Uptake, and NUE

**Figure 4:**
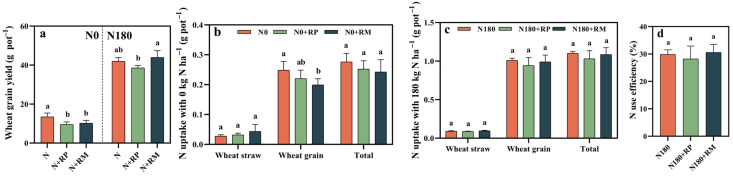
Effects of forest fine root litters derived from *Populus deltoides* (RP) and *Metasequoia glyptostroboides* (RM) on the wheat grain yield (**a**), N uptake capacity (**b**,**c**), and N use efficiency (**d**) from no N (N0) and 180 kg N ha^−1^ (N180) applied to farmland soils. Different lowercase letters indicate that there were significant (*p* < 0.05) differences between the treatments with same N application rate by the Duncan’s method.

Forest fine root litter amendments significantly (*p* < 0.05) decreased the wheat grain yield by 24.4–28.9% without N treatment (Figure 4a). When we applied 180 kg N ha^−1^, the response of wheat grain yield to forest fine root litter was dependent on forest type, though the differences were not statistically significant (*p* ≥ 0.05). The wheat yield of N180+RP was lower than that of N180 alone, while N180+RM produced 4.5% more wheat grain than N180. In addition, almost no significant change was observed in total N uptake by wheat after fine root litter amendment, except that the grain N uptake from N0+RM was 20.0% lower than that from N0 alone (Figure 4b). Although there were no significant differences in wheat NUE between fine root litter additions and no fine root litter additions, the maximum of wheat NUE occurred in N180+RM with 30.6% (Figure 4d).

## 3. Discussion

### 3.1. Effect of Forest Fine Root Litter on NH_3_ Volatilization

The soil pH, NH_4_^+^-N content, and urease activity are always considered to be vital driving factors to NH_3_ volatilization [20,31], and these factors might be altered following the addition of forest fine root litters. The current study identified that forest fine root litters decreased the NH_3_ volatilization from cropland soils with 180 kg N ha^−1^ (Figure 1). Fine root litter reduced the soil NH_4_^+^-N content at both observations whether N was applied or not (Figure S2a,e); although the variation in them was not significant, it was enough to explain the changes in NH_3_ volatilization (12th day after BF). Cui et al. (2021) demonstrated a positive correlation between soil NH_4_^+^-N content and NH_3_ volatilization [31], which could explain the decline of NH_3_ volatilization flux. Additionally, soil urease activity is also positively related to NH_3_ volatilization [20]. Previous field experiments confirmed that a poplar–wheat intercropping system significantly increased soil enzyme activities including urease compared with a monoculture wheat system [32]. However, in the current work, forest fine root litters significantly (*p* < 0.05) decreased soil urease at the ninth day after the BF with 180 kg N ha^−1^ was applied and decreased at the eighth day after the SF with no fertilizer (Figure S2d,h). Meanwhile, more studies should be conducted under natural field conditions to obtain a better understanding of the reduction of NH_3_ volatilization. Fine root litter addition in this study was like the study that showed that exogenous additives such as straw and biochar reduced NH_3_ volatilization as the result of decreased soil urease activity [33].

Moreover, Lei et al. (2021) [34] provided comprehensive insights into the microbiological perspective that *amoA* and *nirS* genes had negative correlations with NH_3_ volatilization. In particular, *amoA* was regarded as a marker of the soil NH_4_+-N oxidation reaction [4]. According to Table 1, there were both AOB *amoA* gene copies during the BF and *nirS* gene copies during the SF observation period under N180+RM, explaining the inhibition of RM on NH_3_ volatilization. Nevertheless, the reduction of AOA and AOB *amoA* as well as *nirS* gene copies in the N180+RP treatment indicated that N functional genes were not the dominant factors controlling the effect of RP on NH_3_ volatilization. Of course, an index should be determined and a model could be constructed to comprehensively understand the relationship between soil physiochemical and microbial properties and NH_3_ volatilization [15,35]. Overall, forest fine root litters regulated NH_3_ volatilization by altering soil NH_4_^+^-N content, urease activity, and AOA and AOB *amoA* gene copies, especially at the BF observation, corresponding to the conclusion that NH_3_ volatilization was concentrated in the BF throughout the whole wheat season. Treatments with lower NH_3_ volatilization help to manage the agroforestry ecosystem’s sustainability.

### 3.2. Changes in Farmland Soil N_2_O Emission as a Result of Forest Fine Root Litters

Forest fine root litters mitigated the total N_2_O emission throughout the whole wheat season, whether urea N fertilizer was applied or not (Figure 2). There was also a strong correlation between N_2_O emissions and functional gene abundances of AOA and AOB *amoA*, *nirK*, *nirS*, and *nosZ*, which were involved in nitrification and denitrification processes [31,34]. The gene copies of *nirS* and *nirK* had positive correlations with N_2_O in previous research [34]. However, this research suggested that fine root litter suppressed the N_2_O emissions though they amplified soil *nirS* and *nirK* gene copies during the BF observation, except in the N180+RP treatment (Table 1). According to Bakken et al. (2012), increasing *nosZ* gene copies could also reduce N_2_O emission by synthesizing more N_2_O reductase [36]. Both fine root litters enhanced the abundances of the *nosZ* gene during the SF at 0 and 180 kg N ha^−1^ application (Table 1). Meanwhile, the same results were also recorded in the BF observation with no fertilizer added. The elevated soil cumulative N_2_O emissions were due to the higher AOB and AOA gene abundances with soil N application [37]. Likewise, forest fine root litters lowered both AOA and AOB *amoA* gene copies of soil with N at 180 kg ha^−1^, which was positively related to N_2_O emissions (Table 1). Therefore, we suggested that the changes in AOA and AOB *amoA* as well as *nosZ* were the major indicators for the influence of fine root litter on N_2_O emissions. More factors determining the N_2_O generated and emitted from cropland soil impacted by forest fine root litters should be clarified in future research.

In addition, the changes in soil physiochemical properties caused by forest fine root litter underlined the decreased N_2_O emissions from fine root litter-added treatments. Firstly, whether N was applied or not, lower soil bulk density (SBD) was recorded following the fine root litters (Figure 3e). Cheng et al. (2016) documented that SBD was significantly positively correlated with N_2_O emission [38]. In addition, SBD was negatively correlated to fine root biomass stocks and decomposition, corresponding to this study’s finding that forest fine root litter input reduced the SBD [21]. Moreover, Bakken et al. (2012) demonstrated that a low pH limited the activity of N_2_O reductase, inhibiting the conversion of N_2_O to N_2_, and thus increasing N_2_O emission [36]. Therefore, slightly elevated soil pH caused by fine root litter may be another rational explanation for the reduction of N_2_O emissions by the corresponding treatments (Figure 3f). In addition, fine root litter significantly decreased soil NO_3_^−^-N content during the SF (Figure S2b,f) and the harvest period (Figure 3b), which not only delayed the nitrification process, but also emphasized that the substrate for denitrification also decreased [31,39]. The variation tendency of soil NH_4_^+^-N content was the same as the N_2_O emission in the fine root litter amended treatments with N fertilizer addition. It was consistent with the viewpoint that lower soil NH_4_^+^-N could decrease the substrates for nitrification and denitrification, resulting in less N_2_O emission [40]. Furthermore, for no N-fertilizer-applied soil, fine root litter input increased the SOM (Figure 3i), which improved soil C/N and then might decrease the N_2_O emission. As reported, the relationship between C/N and N_2_O emission is actually complex and non-linear. High C/N ratios can either increase or decrease N_2_O depending on oxygen availability (aerobic vs. anaerobic conditions), soil moisture content, microbial community composition, labile vs. recalcitrant C fractions, N availability, and immobilization dynamics [41], which could be explored in a future study.

### 3.3. Responses of Wheat Production and NUE to Forest Fine Root Litter

Previous research observed that there was a negative effect of agroforestry ecotone on crop yield due to the canopy shading and competition of water between the crop and living forest fine roots [30], but it is unclear whether forest root litter will simultaneously contribute to the reduction of crop yield. In this study, fine root litter application decreased the wheat yield without N fertilizer, and thereby we hypothesized that fine root litters may also limit grain yield without sufficient nutrients. However, Scordia et al. (2023) pointed out that the response of crop yield was related to the tree species and management synergies [42]. A study revealed that the amount of wheat yield was compared as follows: sole crop system>poplar–wheat system> eucalyptus–wheat system [12]. Consistently, when no fertilizer was applied in this study, both fine root litters significantly (*p* < 0.05) lowered the wheat grain yield. Under the 180 kg N ha^−1^ addition, although there were no significant differences between fine root litter and no fine root litter, RP decreased the wheat yield and RM increased the wheat yield (Figure 4a). These results exactly indicated that appropriate tree species and N management in agroforestry ecosystems do not exert negative impacts on crop production, providing theoretical support for agroforestry system management. The changes in crop yield were attributed to the variations in spike number, kernels per spike, and the thousand kernels weight [43]. Fine root litters were responsible for yield reduction due to the decreases in kernels per spike and the thousand grain weights without N fertilizer input. In addition, the wheat plant height significantly decreased at earing and maturation stage, providing evidence of yield reduction (Figure S1), which was consistent with previous work [18]. Considering food security, it was interesting that N180+RM created more wheat grain than N180 alone, owing to the increasing spike numbers (+8.1%) (Table S2), and Dou et al. (2024) also found this [44]. In addition, the harvest index in N180+RM also increased, although there were no statistically significant differences in the thousand kernels weight and harvest index among all experimental treatments under the same N application level (Table S2). In conclusion, rationally applying N fertilizer and choosing tree species should be the essential pathways for addressing problems in agroforestry systems.

Previous work has assumed that organic additives (such as organic manure and crop residue) enhanced N use efficiency [45]. Dead forest fine root as forest litter was also a dominant organic matter, and may affect N absorption and utilization by decomposition and production [46]. Our data demonstrated that fine root litter promoted N content for the no-N-added groups, whereas it did not affect N content and N uptake when the N-applied rate reached 180 kg ha^−1^ (Table S2 and Figure 4). This consequence suggested that forest fine root litters had a more remarkable influence on the N uptake and utilization when N fertilizer was inadequate. At the same time, two fine root litter species presented different effects on the wheat NUE, in which N180+RP promoted the NUE but N180+RM decreased it. The relationship between plant roots and N uptake was different in various plant species, so the choice of tree species was critical for the crop NUE in agroforestry systems [47]. Although fine root litters exerted a risk of yield reduction, appropriate tree species and fertilizer application (N180+RM) could elevate the wheat production. Meanwhile, forest fine root litter significantly decreased environmental N lost via NH_3_ volatilization and N_2_O emission. In conclusion, choosing appropriate tree species (*Metasequoia glyptostroboides*) will reduce environmental problems while enhancing crop production in agroforestry regions.

This study comprehensively suggested that the incorporation of fine root litters significantly reduced the gaseous N emissions via NH_3_ and N_2_O without crop yield loss from N-added farmland soil in agroforestry ecosystems. Although forest fine root litter causes a wheat grain yield decrease under the no-N-added soil, there was no statistically significant difference with 180 kg N ha^−1^ applied to the soil. In addition, the appropriate tree species (*Metasequoia glyptostroboides*) will slightly elevate the wheat grain yield. However, the research was designed as a soil pot simulation experiment, which differs from the agroforestry system of the field. Furthermore, larger scale and more types of agroforestry need to be carried out to assess the crop production and environmental efficiency.

## 4. Materials and Methods

### 4.1. Background Information on the Pot Experiment

The simulating soil pot experiment was conducted at the Jiangsu Academy of Agricultural Sciences (32°08′ N, 118°82′ E), located in Nanjing, China, with a subtropical monsoon climate, an average 1100 mm annual precipitation, and an average 15.4 °C annual temperature. Soil for the current work was collected from the 0 to 50 cm layer (topsoil from 0 to 20 cm and subsoil from 20 to 50 cm) in the natural field, then was homogenized and dried under air conditions, and subsequently sieved through a 2.0 mm mesh nylon sieve. These soils were repacked into PVC columns with a 30.0 cm inner diameter and 60.0 cm height with consistent soil profiles and the same bulk density (35.0 kg soil per pot). Forest fine root litters with less than 2.0 mm diameter of common agroforestry tree species (i.e., *Populus deltoides* and *Metasequoia glyptostroboides*) in the field were chosen as experimental material [48]. Fine root litter was broken into less than 1.0 cm length sections, and then air-dried naturally. A total of 10.0 g broken fine root litters were mixed with the top (0–20 cm) soils to simulate forest fine roots entering agricultural soil as would result from field management practices such as ploughing in an agroforestry system. The experimental soil was classified as the Hydragric Anthrosol and the characteristics of the 0–20 cm topsoil were pH (H_2_O:soil = 2.5:1) 6.89, NH_4_^+^-N 1.13 mg kg^−1^, NO_3_^−^-N 0.35 mg kg^−1^, available P 23.0 mg kg^−1^, available K 124.7 mg kg^−1^, and organic matter 8.44 g kg^−1^.

### 4.2. Experimental Design and Fertilization Practices

Overall, two forest fine root litters (RP and RM) and two N application rates (0 and 180 kg ha^−1^) were considered. No forest fine root litter treatments were applied to the control. Therefore, a total of six treatments were established and they were abbreviated as N0, N0+RP, N0+RM, N180, N180+RP, and N180+RM, respectively, each with four replicates. Urea (46% N), calcium superphosphate (12% P_2_O_5_), and potassium chloride (60% K_2_O) all in granular forms of were used to provide N, P, and K nutrients for wheat growth, respectively. For 180 kg N ha^−1^ (equivalent of 1.27 g pot^−1^) treatments, the urea was split at ratios of 40% and 60% as the basal (BF, incorporated) and supplemental fertilization (SF1, broadcasted and irrigated immediately) on 11 December 2022 and 19 March 2023, respectively. We incorporated the P and K fertilizers at a one-time application with rates of 90 kg P_2_O_5_ ha^−1^ and 200 kg K_2_O ha^−1^, respectively (the equivalent of 0.75 and 1.50 g pot^−1^), as BF. Wheat (*Triticum aestivum* L., var. Ningmai 12) was sown with 50 seeds in each soil pot on 11 December 2022, and then thinned to 30 seedlings per soil pot on 7 February 2023. The wheat was harvested on 17 May 2023. Manual weeding and appropriate irrigation were conducted throughout the wheat growing period according to local farmers’ traditional practices. We irritated the wheat plants according to their growing demand and the soil water-holding capacity. Therefore, there was no waterlogging during the wheat season experiment.

### 4.3. Samplings and Measurements

#### 4.3.1. Soil NH_3_ Volatilization

The NH_3_ volatilization rate was measured during the 18 days after two N fertilizations, using the continuous airflow enclosure method as described by Min et al. (2021) [49]. The chamber (15.0 cm inner diameter and 20.0 cm height) was the main part of the measurement system, which related to the absorbent bottle with 80 mL of 2% boric acid. The sampling time was 9:00−11:00 and 14:00−17:00 daily, with an airflow rate of 20 chamber volumes per minute, and then titrated with diluted H_2_SO_4_ (0.02 mol L^−1^) every three days. The H_2_SO_4_ volume was recorded to calculate the NH_3_ volatilization rate. The total NH_3_ loss was the sum of NH_3_ volatilization within the 18 days after fertilization at each fertilizer period. The emission factor and yield-scale NH_3_ volatilization were calculated according to the following formulas [50]:

$$\frac{\mathrm{Emission}\;\mathrm{factor}\;(\%)={\mathrm{NH}}_{3}\;\mathrm{volatilization}\;\mathrm{in}\;N\;\mathrm{added}\;\mathrm{treatment}\;(g)\;-\;{\mathrm{NH}}_{3}\;\mathrm{volatilization}\;\mathrm{in}\;\mathrm{control}\;\mathrm{without}\;N\;\left(g\right)}{\mathrm{the}\;\mathrm{amount}\;\mathrm{of}\;N\;\mathrm{applied}\;(g)}\;\times \;100\%\;\mathrm{Yield-scaled}\;{\mathrm{NH}}_{3}\;\mathrm{volatilization}\;(g\;\mathrm{kg}-1)\;=\;\frac{\mathrm{total}\;{\mathrm{NH}}_{3}\;\mathrm{volatilization}s\;(g)}{\mathrm{wheat}\;\mathrm{grain}\;\mathrm{yield}\;(\mathrm{kg})}$$

#### 4.3.2. N_2_O Emission

A modified closed chamber method was used to collect gas samples for N_2_O determination [50]. A transparent Plexiglas cylinder was applied as a closed chamber (36.0 cm inner diameter and 100.0 cm height) sealed within the pot using water in a recess. For each sampling, four gas samples were taken by a syringe at 0, 15, 30, and 45 min after the chamber was sealed by water. The sampling times were the 3rd, 6th, 9th, and 12th days after each fertilization period and every 14 days throughout the other periods of wheat growth until harvest. The N_2_O concentration of the gas sample was immediately determined with a gas chromatograph (Agilent 7890B, Agilent Technologies, Santa Clara, CA, USA, with a precision of ± 0.3%) equipped with a ^63^Ni electron capture detector (ECD) and back-flush controlled by a 10-port value in the laboratory. The N_2_O emission flux was calculated by the formula below [50]:

$$N_{2}O\;\mathrm{emission}\;\mathrm{flux}\;(µg\;m-2\;h-1)\;=\;\rho \;\times \;h\;\times ∆c/∆t\times 273/T$$

ρ indicates the density of N_2_O under standard conditions (1.25 kg m^−3^). h represents the height of the transparent Plexiglas cylinder (m), and 100.0 cm converts to 1.0 m. ∆c/∆t refers to the changing rates in the gas concentration of the gas sample in the cylinder over a specific time interval (µg L^−1^ h^−1^), and 60 min converts to one hour. T denotes the recorded temperature inside of the cylinder (Kelvin). The cumulative N_2_O emission was the sum of daily N_2_O emission fluxes.

The yield-scaled N_2_O emission and emission factor were calculated according to the formulas below [50]:

$$\mathrm{Yield-scaled}\;N_{2}O\;\mathrm{emission}\;(g\;\mathrm{kg}-1)\;=\;\frac{\mathrm{total}\;N_{2}O\;\mathrm{emission}\;(g)}{\mathrm{grain}\;\mathrm{yield}\;(\mathrm{kg})}$$

$$\mathrm{Emission}\;\mathrm{factor}\;\left(\%\right)=\frac{N_{2}O\;\mathrm{emission}\;\mathrm{in}\;N\;\mathrm{added}\;\mathrm{treatment}\;(g)\;-\;N_{2}O\;\mathrm{emission}\;\mathrm{in}\;\mathrm{control}\;\mathrm{without}\;N\;\left(g\right)}{\mathrm{the}\;\mathrm{amount}\;\mathrm{of}\;N\;\mathrm{application}\;(g)}\;\times \;100\%$$

Note: The N_2_O emission in the control with N refers to the N_2_O emission from N0.

#### 4.3.3. Determination of Soil Properties

The 0−20 cm topsoil samples were collected twice during each fertilization stages (i.e., on 20 December and 26 December 2022 during the BF, and on 27 March and 6 April 2023 during the SF1), and once after the wheat harvest (i.e., on 20 May 2023). The soil bulk density was determined using the core method. The contents of soil NO_3_^−^-N, NH_4_^+^-N, and total N were determined by an ultraviolet spectrophotometer, indophenol blue colorimetry, and the Kjeldahl method, respectively. Soil available P and K contents were measured with the molybdenum antimony anti-chromogenic and NH_4_^+^-N acetate extraction-flame photometry methods, respectively [51]. Sodium phenol-sodium hypochlorite colorimetry was used to determine soil urease activity [52].

In order to assess the functional gene abundances that related to soil N cycling including the *amoA* genes of ammonia-oxidizing archaea and bacteria (i.e., AOA *amoA* and AOB *amoA*, respectively), *nirS*, *nirK*, and *nosZ*, a real-time quantitative polymerase chain reaction (qPCR) was conducted [53]. The measurement was provided by Beijing Qingke Biotechnology Co., LTD. The first step was to extract soil DNA with the magnetic bead soil and fecal Genomic DNA extraction kit (DP712, TIANGEN Biochemical Technology, China) from fresh soil samples. Then, qPCR reactions were performed to determine the copies of the above genes using the Quant StudioTM1 Plus real-time fluorescence quantitative PCR system (T100 Thermal Cycler, Bio-Rad Laboratories, Inc., USA). The qPCR used the following conditions: 95 °C for 5 min, and then continued for 40 cycles (95 °C for 15 s, 55−60 °C for 20 s, AOA and AOB *amoA*, *nirS*, *nirK*, and *nosZ* at 55 °C, 55 °C, 58 °C, 58 °C, and 60 °C, respectively, and then 72 °C for 20 s), capturing fluorescence in the melt phase. The specific primers for the aforementioned processes are shown in Table S1.

#### 4.3.4. Wheat Growth, Grain Yield, N Uptake, and NUE

We used a tapeline and a SPAD 502 m (Konica Minolta, Tokyo, Japan) to determine the wheat plant height and soil and plant analyzer development (SPAD) values two times at the jointing and earing stages during the wheat growth season, respectively. Wheat agronomic traits (including straw dry biomass, grain yield, spike number, and kernels per spike as well as thousand kernels weight) were recorded after we harvested the straw and grain separately. The total N content of straw and grain was measured with the Kjeldahl method by Lu (2000), i.e., digesting the crushed plant samples with H_2_SO_4_ and H_2_O_2_ [51]. The harvest index, N uptake, and NUE were calculated according to the following formulas [50]:

$$\mathrm{Harvest}\;\mathrm{index}\;\left(\%\right)\;=\;\frac{\mathrm{Grain}\;\mathrm{dry}\;\mathrm{weight}\;(g)}{\mathrm{Grain}\;\mathrm{dry}\;\mathrm{weight}\;(g)\;+\;\mathrm{straw}\;\mathrm{dry}\;\mathrm{weight}\;(g)}\;\times \;100\%$$

Straw/grain N uptake (g)=Dry weight of straw/grain (g) × N content of straw/grain (g kg−1) / 1000

$$\mathrm{NUE}\;\left(\%\right)=\frac{N\;\mathrm{uptake}\;\mathrm{in}\;N\;\mathrm{added}\;\mathrm{treatment}\;(g)\;-\;N\;\mathrm{uptake}\;\mathrm{in}\;\mathrm{control}\;\mathrm{without}\;N\;(g)}{N\;\mathrm{application}\;\mathrm{rate}\;(g)}\;\times \;100\%$$

### 4.4. Statistical Analysis

We used Excel 2021 and IBM SPSS 22 (SPSS Inc., Chicago, IL, USA) to process data and perform a one-way ANOVA of variation. Figures were created using GraphPad Prism 8.0.2. The significant difference between treatments under the same N application levels were determined according to Duncan’s multiple range test at a *p* < 0.05 significance level.

## 5. Conclusions

We conducted a pot/column experiment to simulate the effects of two forest fine root litters on gaseous N losses and wheat grain yield in farmland soil collected from natural conditions. Both forest fine root litters had little impact on the total NH_3_ volatilization from farmland soils with no N fertilizer applied. With 180 kg N ha^−1^ applied to the soil, forest fine root litter significantly (*p* < 0.05) reduced the total NH_3_ volatilization and its emission factor. The decline in NH_3_ volatilization was correlated with the reduction of soil NH_4_^+^-N content and urease activity. Meanwhile, the soil AOA and AOB *amo*A gene copies also decreased following the application of forest fine root litter. Forest fine root litter effectively decreased the total N_2_O loss and the yield-scaled N_2_O emission in the whole wheat season whether N fertilizer was applied or not, while it did not affect the emission factor with 180 kg N ha^−1^ applied soils. The N_2_O emission was negatively related to soil pH and *nosZ* gene copies, but positively linked to the soil bulk density, NH_4_^+^-N, NO_3_^−^-N, and the soil AOA and AOB *amoA* gene copies. The wheat yields were not influenced significantly by the occurrence of forest fine root litter, and N180+RM even produced more grain yield than N180. Therefore, appropriate N application and tree species could effectively mitigate N losses via NH_3_ and N_2_O, without influencing stable crop production.

## Figures and Tables

**Table 1:** Effects of forest fine root litters of *Populus deltoides* (RP) and *Metasequoia glyptostroboides* (RM) on the N cycling-related functional gene copies in 0−10 cm topsoil sampled at the BF and SF stages from the farmland with 0 (N0) and 180 kg N ha^−1^ (N180) application.

Fertilization Period	Treatment	AOA *amoA*	AOB *amoA*	*nirK*	*nirS*	*nosZ*	*nirS*+*nirK*/*nosZ*
		10^6^ Copies g^−1^	10^7^ Copies g^−1^	10^8^ Copies g^−1^			
BF	N0	3.42 ± 0.40 b	0.87 ± 0.20 a	1.48 ± 0.24 a	2.61 ± 0.68 c	2.06 ± 0.29 b	1.99 ± 0.38 a
	N0+RP	2.69 ± 0.38 b	0.79 ± 0.19 a	1.67 ± 0.20 a	3.62 ± 0.65 b	2.73 ± 0.57 ab	2.00 ± 0.48 a
	N0+RM	4.65 ± 0.55 a	0.89 ± 0.41 a	1.94 ± 0.51 a	3.99 ± 1.04 a	3.33 ± 0.65 a	1.86 ± 0.68 a
	N180	3.19 ± 0.52 a	7.98 ± 0.64 a	1.83 ± 0.14 b	4.11 ± 0.34 a	3.44 ± 0.34 a	1.74 ± 0.23 b
	N180+RP	2.36 ± 0.25 b	6.11 ± 1.35 b	1.65 ± 0.05 b	3.54 ± 0.78 a	2.59 ± 0.36 b	2.00 ± 0.09 ab
	N180+RM	2.55 ± 0.19 a	9.13 ± 1.26 a	2.69 ± 0.22 a	4.07 ± 0.59 a	2.79 ± 0.53 ab	2.50 ± 0.57 a
SF	N0	2.80 ± 0.55 a	1.54 ± 0.08 b	1.67 ± 0.18 c	1.23 ± 0.16 c	3.63 ± 0.97 a	0.83 ± 0.20 b
	N0+RP	2.32 ± 0.37 a	2.01 ± 0.19 a	1.99 ± 0.11 b	2.74 ± 0.40 b	4.01 ± 1.01 a	1.23 ± 0.30 ab
	N0+RM	2.17 ± 0.44 a	1.73 ± 0.09 b	3.80 ± 0.27 a	4.68 ± 0.67 a	5.32 ± 1.54 a	1.66 ± 0.32 a
	N180	3.02 ± 0.62 a	32.05 ± 3.86 a	3.33 ± 0.81 a	3.96 ± 1.00 a	4.16 ± 1.37 b	1.82 ± 0.29 a
	N180+RP	2.36 ± 0.84 a	21.08 ± 6.04 b	4.04 ± 0.84 a	3.97 ± 0.09 a	5.57 ± 1.31 ab	1.48 ± 0.30 a
	N180+RM	2.46 ± 0.59 a	28.11 ± 0.75 a	4.27 ± 1.07 a	5.19 ± 1.30 a	6.98 ± 1.17 a	1.37 ± 0.22 a

Note: BF and SF refer to the basal and supplementary N fertilization, respectively. Data are mean ± SD (*n* = 4). Different lowercase letters indicate that the differences between the treatments with the same N application rate were statistically significant by Duncan’s method at *p* < 0.05.

## Data Availability

The original contributions presented in this study are included in the article/Supplementary Materials. Further inquiries can be directed to the corresponding authors.

## Supplementary Materials

The following supporting information can be downloaded at https://www.mdpi.com/article/10.3390/plants15010057/s1. Figure S1: Effects of forest fine root litter of *Populus deltoides* (RP) and *Metasequoia glyptostroboides* (RM) on the wheat plant height (**a**,**b**) and flag leaf SPAD value (**c**,**d**) at different wheat growth stages from 0 (N0) and 180 kg N ha^−1^ (N180) applied farmland soils. Different lowercase letters indicate the significant differences between the treatments with same N application rate by Duncan’s method at *p* < 0.05 significance level. Figure S2: Effects of forest fine root litter of *Populus deltoides* (RP) and *Metasequoia glyptostroboides* (RM) on the top layer (0−10 cm) soil properties during the BF and SF from 0 (N0) and 180 kg N ha^−1^ (N180) applied farmland soils. BF and SF refer to the basal and supplementary N fertilization, respectively. Different lowercase letters indicate the significant differences between the treatments with same N application rate by Duncan’s method at *p* < 0.05 significance level. Table S1: Primers for real-time PCR quantification of AOA *amo*A, AOB *amo*A, *nir*S, *nir*K, and *nos*Z genes. Table S2: Responses of the yield-related agronomic traits and N content of wheat to forest fine root litter of *Populus deltoides* (RP) and *Metasequoia glyptostroboides* (RM) from 0 (N0) and 180 kg N ha^−1^ (N180) applied farmland soils. References [54,55,56] are cited in the supplementary materials.
