# Supplementary material for: Forest Fine Root Litter Mitigates the NH3 Volatilization and N2O Emission from N-Applied Agriculture Soil
Source: Plants (Basel). 2025 Dec 24;15(1):57. doi: 10.3390/plants15010057 (PMC12788022; doi:10.3390/plants15010057)
Supplement: Supplementary file 1 [file plants-15-00057-s001.zip › plants-4013918-supplementary.pdf]

Supplementary materials

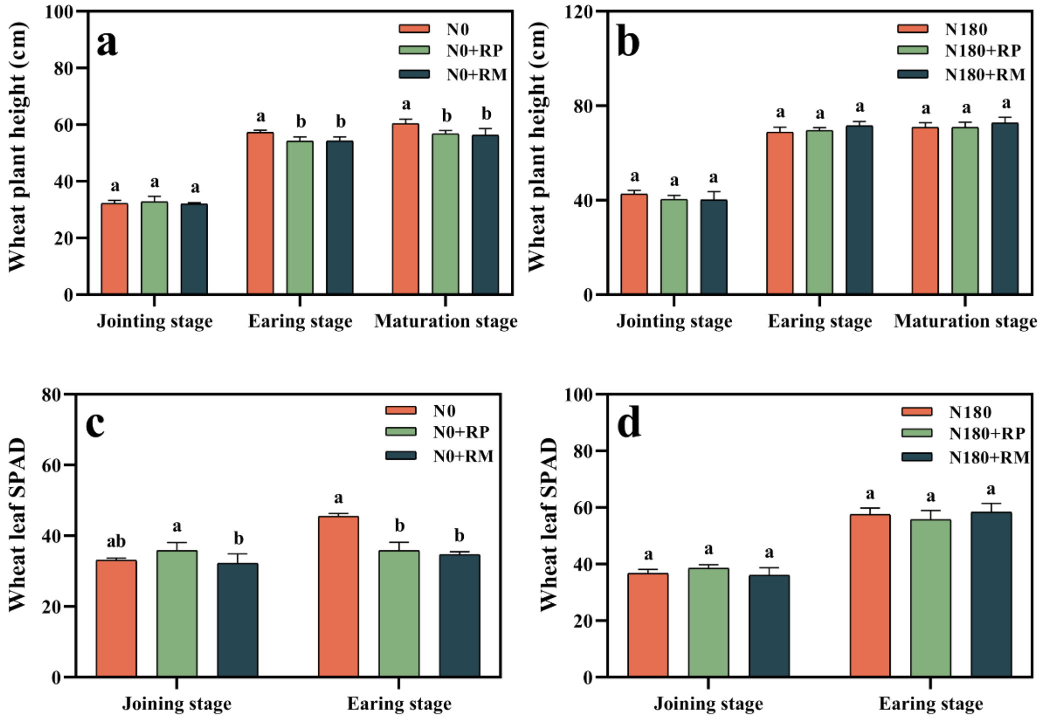

Figure S1. Effects of forest fine root litter of *Populus deltoides* (RP) and *Metasequoia glyptostroboides* (RM) on the wheat plant height (a, b) and flag leaf SPAD value (c, d) at different wheat growth stage from 0 (N0) and 180 kg N ha^{-1} (N180) applied farmland soils. Different lowercase letters indicate the significant differences between the treatments with same N application rate by Duncan's method at $p < 0.05$ significance level.

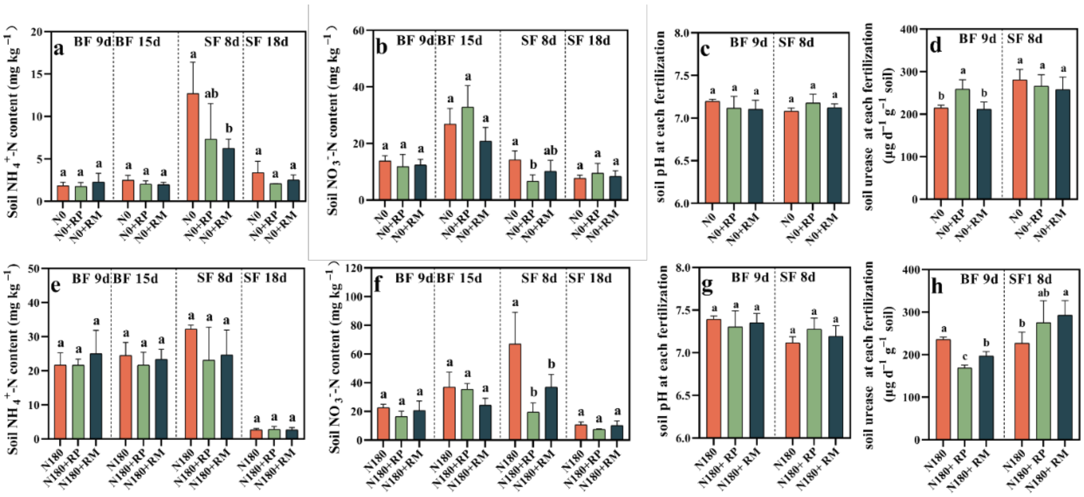

Figure S2. Effects of forest fine root litter of *Populus deltoides* (RP) and *Metasequoia glyptostroboides* (RM) on the top layer (0–10 cm) soil properties during the BF and SF from 0 (N0) and 180 kg N ha^{-1} (N180) applied farmland soils. BF and SF referred to the basal and supplementary N fertilization, respectively. Different lowercase letters indicate the significant differences between the treatments with same N application rate by Duncan's method at $p <$ 0.05 significance level.

Table S1. Primers for real-time PCR quantification of AOA *amoA*, AOB *amoA*, *nirS*, *nirK* and *nosZ* genes.

Genes	Primers	Sequences (5'-3')	References
nirk	*nirK* 876	ATYGGCGGVCAYGGCGA	Li et al. (2015)
	nirK 1040	GCCTCGATCAGRTTRTGGTT	
AOA*amoA*	Arch-*amo*AF	STAATGGTCTGGCTTAGACG	Tong et al. (2012)
	Arch-*amo*AR	GCGGCCATCCATCTGTATGT	
AOB*amoA*	*amo*A-1F	GGGGTTTCTACTGGTGGT	Chen et al. (2021)
	*amo*A-2R	CCCCTCKGSAAAGCCTTCTTC	
nirS	*nirS* Cd3aF	AACGYSAAGGARACSGG	Li et al. (2015)
	nirS R3cd	GASTTCGGRTGSGTCTTSAYGAA	
nosZ	*nos*Z1F	WCSYTGTTCMTCGACAGCCAG	Shan et al. (2021)
	*nos*Z1R	ATGTCGATCARCTGVKCRTTYTC	

References:

1. Li, X., Wang, H., Hu, C., Yang, M., Hu, H.Y., Niu, J.F., 2015. Characteristics of biofilms and iron corrosion scales with ground and surface waters in drinking water distribution systems. Corros. Sci. 90, 331-339.

2. Shan, J., Sanford, R.A., Chee-Sanford, J., Ooi, S.K., Löffler, F.E., Konstantinidis, K.T., Yang, W.H., 2021. Beyond denitrification: the role of microbial diversity in controlling nitrous oxide reduction and soil nitrous oxide emissions. Global Change Biol. 27(12), 2669-2683.

3. Chen, Z.J., Jin, Y.Y., Yao, X., Wei, X.K., Li, X.Z., Li, C.J., White, J.F., Nan, Z.B., 2021. Gene analysis reveals that leaf litter from Epichloë endophyte-infected perennial ryegrass alters diversity and abundance of soil microbes involved in nitrification and denitrification. Soil Biol. Biochem. 154, 108123. https://doi.org/10.1016/j.soilbio.2020.108123.

4. Tong D, Xu R., 2012. Effects of urea and $(NH_4)_2SO_4$ on nitrification and acidification of Ultisols from Southern China. J. Environ. Sci. 24(4), 682-689.

Table S2. Responses of the yield related agronomic traits and N content of wheat to forest fine root litter of *Populus deltoides* (RP) and *Metasequoia glyptostroboides* (RM) from 0 (N0) and 180 kg N ha^{-1} (N180) applied farmland soils.

Treatment	Spike number	Kernels per spike	Thousand kernels weight (g)	Harvest index (%)	Wheat grain N content (g kg^{-1})
N0	25±1 a	12±1 a	46.0±2.3 a	55.2±4.3 a	18.53±0.76 b
N0+RP	26±1 a	9±1 b	44.0±1.8 a	49.0±6.4 a	23.16±2.68 a
N0+RM	27±2 a	9±2 b	44.2±3.9 a	52.4±4.4 a	19.58±2.21 b
N180	37±3 a	26±2 a	45.2±2.9 a	60.8±1.7 a	24.06±0.52 a
N180+ RP	41±2 a	21±1 b	45.7±1.0 a	60.6±1.9 a	24.41±2.26 a
N180+ RM	40±4 a	25±3 a	44.3±1.3 a	63.0±1.3 a	22.54±0.55 a

Note: Data are shown as Mean ± SD (n = 4). Different lowercase letters indicate the significant differences between the treatments with same N application rate by Duncan's method at $p <$ 0.05 significance level.
